# From flagellar undulations to collective motion: predicting the dynamics of sperm suspensions

**DOI:** 10.1098/rsif.2017.0834

**Published:** 2018-03-21

**Authors:** Simon F. Schoeller, Eric E. Keaveny

**Affiliations:** Department of Mathematics, Imperial College London, South Kensington Campus, London SW7 2AZ, UK

**Keywords:** sperm locomotion, collective dynamics, active suspensions, fluid–structure interactions

## Abstract

Swimming cells and microorganisms are as diverse in their collective dynamics as they are in their individual shapes and propulsion mechanisms. Even for sperm cells, which have a stereotyped shape consisting of a cell body connected to a flexible flagellum, a wide range of collective dynamics is observed spanning from the formation of tightly packed groups to the display of larger-scale, turbulence-like motion. Using a detailed mathematical model that resolves flagellum dynamics, we perform simulations of sperm suspensions containing up to 1000 cells and explore the connection between individual and collective dynamics. We find that depending on the level of variation in individual dynamics from one swimmer to another, the sperm exhibit either a strong tendency to aggregate, or the suspension exhibits large-scale swirling. Hydrodynamic interactions govern the formation and evolution of both states. In addition, a quantitative analysis of the states reveals that the flows generated at the time scale of flagellum undulations contribute significantly to the overall energy in the surrounding fluid, highlighting the importance of resolving these flows.

## 1. Introduction

Swimming cells and microorganisms encompass the entire range of cell types and exhibit a great variety of cell geometries and swimming strategies used to move in viscosity dominated environments [1,2]. Some organisms, such as *Volvox* colonies, can be nearly spherical and use for propulsion thousands of relatively short flexible flagella distributed along their surface. Others, like sperm cells, have a single, long flagellum that propagates bending waves, leading to a large time-periodic shape change of the cell. Though stereotyped, the propagated waveforms, frequencies and head shapes of sperm cells vary appreciably across different species [3].

There is not only great variation among individual cells, but also in the patterns that they form and in how populations are organized. This can range from algal plume formation in bioconvection [4,5] to turbulence-like swirling in bacterial baths [6–9]. Great variation is exhibited by sperm suspensions of different species that have been observed to form vortices near planar boundaries [10], aggregate into coherent sperm trains [11] or exhibit turbulence-like swirling as documented nearly 70 years ago by Lord Rothschild [12–15]. What is not clear, however, is how the individual differences in sperm cells, such as geometry, flagellum length, waveform and flexibility, give rise to the differences in the way sperm populations organize themselves. Mathematical modelling and simulation provide a route to explore the connection between individual and collective dynamics where experiments might otherwise be very challenging.

In the past 10–15 years, there has been much work on the development of mathematical models [8,16–24] of microorganism suspensions, especially to understand the turbulence-like state found in bacterial baths. To deal with large numbers of cells, the models rely on a reduced description of the swimmers, often treating them as simple, rigid objects (e.g. rods, dumbbells, spheres and ellipsoids) that interact through steady, dipolar flows and steric repulsion. These models have been effective in reproducing the large-scale motion of the population and relating its formation to the sign of the coefficient of dipolar flow induced by individuals.

While such models now broadly shape our thinking about suspensions of swimming microorganisms, they reduce the diversity of the microscopic world into a handful of parameters and a limited number of degrees of freedom. They ignore the complexity and time-dependence of cell shapes, as well as the time-dependent and beyond-dipolar features of the flows generated by the shape changes. Indeed, many of these details have been included in models of individual or small collections of swimmers. In the case of sperm cells, the addition of flagellar motion leads to hydrodynamically induced attraction, synchronization and phase-locking of planar, flagellar waveforms [25–27]. In simulations of 50–100 swimmers [28,29], these effects were found to induce aggregation and clustering of cells. This tendency to aggregate may aid sperm in forming coherent groups such as sperm trains; however, it remains unclear how a turbulence-like state could then be reached.

In this paper, we perform detailed simulations of up to 1000 interacting sperm, resolving the coupled flagellar dynamics along with the flows generated at the undulation time scale and at sub-flagellum length scales. We report that variation in the individual dynamics across the population, here controlled by the undulation frequency, can suppress the tendency to aggregate and instead lead to a density-dependent turbulence-like state with hydrodynamics still playing a key, but now different role. Additionally, an analysis of the clustered and turbulence-like states reveals the strong influence of flagellar undulation on the quantities used to explore large-scale motion in swimmer suspensions. These results suggest that only minor variations in sperm behaviour across species are needed to produce very distinct collective dynamics.

## 2. Mathematical model

Our swimmer model is closely related to several models [30–34] employed to describe the dynamics of sperm cells in a viscous fluid and extends directly from a model previously used to capture undulatory locomotion through structured media [35]. We briefly summarize it here in the context of a single swimmer and provide a more detailed description in the electronic supplementary material.

Our swimmers consist of two elements, the flagellum and the cell head. The flagellum is treated as an inextensible, yet flexible beam of length *l* and bending modulus *K*_B_, while an oblate spheroid of semi-major and minor axes *a* and *b*, respectively, represents the cell head. The overall swimmer length is taken to be *d* = 2.1*a* + *l*, where the distance 0.1*a* accounts for a linkage between the flagellum and the head. The flagellum is parametrized by arclength *s* ∈ [0, *l*] such that the position of a point along the flagellum is **Y**(*s*) and the unit tangent at that point is 
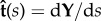
. The flagellum is driven internally by the moments per unit length, ***τ***^*D*^, that arise due to the preferred curvature,2.1
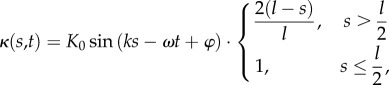
where *ω* = 2*π*/*T* is the undulation frequency, *k* is the wavenumber, *φ* is the phase, and *K*_0_ is the amplitude. The linear decay in the preferred curvature amplitude near the free end is introduced to better reproduce observed flagellar waveforms. Allowing the flagellum to also be subject to external applied forces, **f**, and torques, ***τ***, per unit length due to viscous stresses, the force and moment balances along the flagellum are2.2
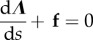
and2.3
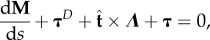
where ***Λ*** is the internal force on flagellum cross sections and 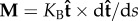 is the bending moment. At one end (*s* = 0), the flagellum is attached via a clamped-end condition to the cell head, while the other end (*s* = *l*) remains free.

The continuous beam equations (2.2) and (2.3) are discretized to obtain force and torque balances on *N*_flag_ segments of the flagellum, as well as the cell head (see the electronic supplementary material for details). For the *n*th segment of the flagellum, the balances are2.4
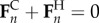
and2.5
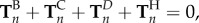
where **T**^B^_*n*_ are torques arising due to bending, **F**^C^_*n*_ and **T**^C^_*n*_ are constraint forces and torques associated with tension, **T**^*D*^_*n*_ are the torques due to the preferred curvature, and **F**^H^_*n*_ and **T**^H^_*n*_ are the hydrodynamic forces and torques on the segment. The force and torque balances yield a low Reynolds number mobility problem for the translational and angular motion of the segments that is identical to that for a collection of rigid particles. We solve this mobility problem using a regularized multipole approach for Stokes flows known as the *force-coupling method* (FCM) [35–39]. In the limit of large *N*_flag_, FCM provides an approximation of the hydrodynamics consistent with a regularized version of slender body theory [31,33,40–44]. After solving the mobility problem for the velocities and angular velocities of the flagellum segments and cell head, the differential equations for their positions and orientations are integrated in time using a second-order backwards differentiation scheme. Broyden's method is then used to solve the resulting system of equations for the updated positions and orientations, and the Lagrange multipliers associated with the forces and torques arising from the inextensibility constraint.

We choose the parameters in our simulations to reproduce a cell geometry, flagellar waveform and swimming speed close to that reported for ram sperm [14,45] and in the range of other mammalian sperm [46,47]. The head size *a* is such that *a*/*l* = 0.0470 and *b* = *a*/3. The wavenumber is *k* = 2*π*/*l*, and the curvature amplitude is *K*_0_ = 12.76/*l*. Finally, we set the dimensionless parameter known as the *sperm number*, which provides a measure of the ratio of the viscous to elastic forces [2,35,48–50], to Sp = (4*πωη*/*K*_B_)^1/4^*l* = 12.0, where *η* is the dynamic viscosity. With these parameters, 23.7 undulation periods are required for the sperm to swim its flagellum length.

We perform three-dimensional simulations in domains of size *L* × *L* × *L*_*z*_ corresponding to two-dimensional periodic thin films with finite thickness *L*_*z*_ = 0.277*d*. The corresponding fluid flow is resolved on a grid of size *N*_*x*_ × *N*_*y*_ × *N*_*z*_. Free surface conditions at *z* = 0 and *z* = *L*_*z*_ are established by restricting the motion of the swimmers to the mid-plane *z* = *L*_*z*_/2 and applying periodic boundary conditions in all three directions. This particular choice in boundary conditions and film thickness corresponds to those commonly employed in experiments on swimming microorganisms [6,16,51,52].

When simulating multiple swimmers, a short-ranged repulsive force between nearly touching segments is included to capture steric repulsion between swimmers (see the electronic supplementary material). The strength of this force at contact is determined by the parameter *F*_S_. This and all simulation parameters, including their values in simulation units, are summarized in table 1.

**Table 1.** Simulation parameters.

parameter	description	value in simulation units
*η*	viscosity	1
Sp	sperm number	12
*K*_0_	curvature amplitude	0.2
*K*_B_	bending modulus	1800
*b*	segment radius and head height	1
*a*	head half axis	3
*d*	swimmer length	70.1
*F*_S_/*d*	steric reference force	15*π*
*N*_*x*,*y*_	grid dimensions (in-plane)	{2048, 3072}
*N*_*z*_	grid dimension (normal)	64
*L*	linear domain size	931.3
*L*_*z*_	domain height	19.40
*N*_flag_	number of flagellum segments per swimmer	29
*N*	number of swimmers	{100, …, 1000}
*N*_*t*_/*T*	time-steps per undulation	300
*σ*_*ω*_/*ω*	relative standard deviation of frequencies	{0, 0.2}

## 3. Time-dependent and higher-order features of the swimmer flow field

**Figure 1.**
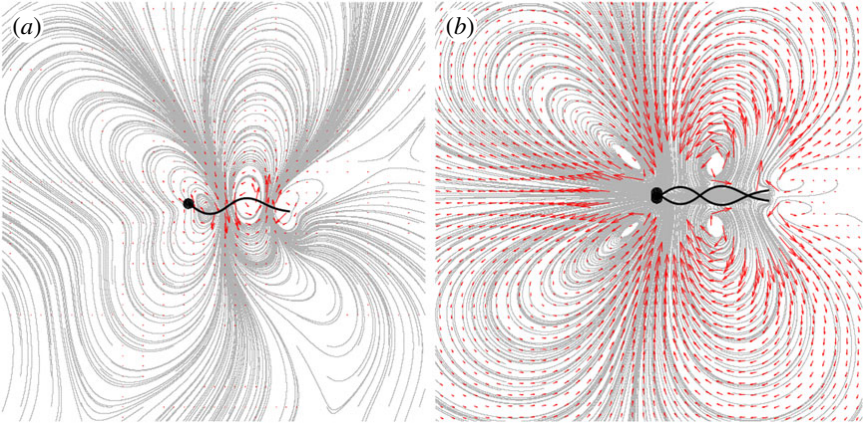
Fluid flow induced by an individual swimmer. (*a*) Instantaneous streamlines (grey) and fluid velocity field (red arrows) at *z* = *L*_*z*_/2 produced by a single swimmer at one instant in time. (*b*) Period-averaged streamlines and fluid velocity field in a co-moving frame at *z* = *L*_*z*_/2. Two snapshots of the swimmer are also shown. (Online version in colour.)

**Figure 2.**
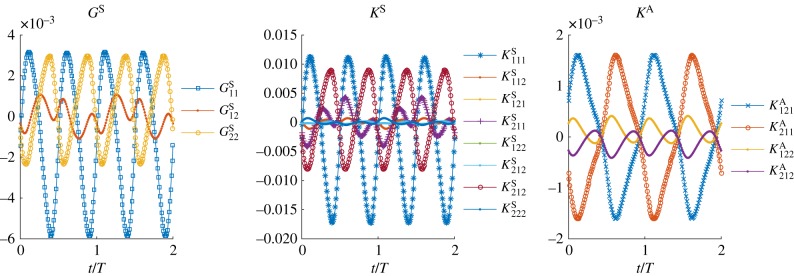
The symmetric, trace-less dipole, *G*_*ij*_, and quadrupole, *K*^S,A^_*ijk*_, coefficients as a function of time. The values are reported in the swimmer frame where the origin is the swimmer's center of mass and 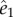 is the average swimming direction. Components that are zero at all times are excluded. Dipole entries are in units of *F*_0_*d*, while the quadrupole entries have units of *F*_0_*d*^2^. (Online version in colour.)

Examining the flow field produced by a single swimmer and the force-moments associated with this flow, we see clear differences between their instantaneous and period-averaged values. The flow at the mid-plane of the thin film and the swimmer at an instant in time are shown in figure 1*a*, while figure 1*b* shows the flow averaged over one undulation period. These flow fields are comparable to those given by other computational studies of individual swimmers propelled by an elastic filament [27–30,53,54]. While the period-averaged flow closely resembles the dipolar flow generated by a so-called pusher, we find that the instantaneous flow field at any point in time is markedly different, containing features of higher-order forces singularities. To analyse this further, we compute (see the electronic supplementary material and [49]) the in-plane dipole and quadrupole force moments for the swimmer as functions of time. The symmetric, trace-less dipole tensor *G*, the symmetric (in the first two indices), trace-less quadrupole tensor *K*^S^ and the anti-symmetric quadrupole tensor *K*^A^ are plotted over two undulation periods in figure 2. The time periodic nature of the force moments, especially their change in sign over the course of a period, is consistent with experimental measurements [51,54,55] on flagellate microswimmers. The anti-symmetric dipole moment is always zero, as there is no net torque on the swimmer. From these time series, we can extract the non-vanishing time-averages, the largest of which are the dipole coefficients 〈(*G*^S^_11_, *G*^S^_22_)〉 = (−1.050, 0.432) × 10^−3^*F*_0_*d*, and the quadrupole coefficients 〈(*K*^S^_111_, *K*^S^_221_)〉 = (−2.321, 0.822) × 10^−3^*F*_0_*d*^2^, where *F*_0_ is the in-plane drag on the cell head moving at the average swimming velocity. We note that for both the dipole and quadrupole these average values are much smaller than the maximum values attained during an undulation period. Quadrupole components (*K*^S^_111_ and *K*^S^_221_) are indeed expected to contribute substantially to the fluid flow around spermatozoa [33,56] and have also been linked to swimmer velocity correlations in bacterial suspensions [57]. The maximum value of the dipole coefficient in our model is more than a factor of 5.5 greater than its mean value, while for the quadrupole this increases to a factor of 7. Additionally, the relatively large values of the quadrupole coefficients produce strong flows that, although they decay faster than the dipolar flow, are non-negligible for significant distances from the swimmer. We estimate that the instantaneous quadrupolar flow dominates the dipolar flow up to separations of approximately 3*d*.

From this, a picture emerges that not only are the time-dependent aspects of the dipolar flow much stronger than the average flow, but also that higher-order contributions are non-negligible when time dependence is included. Both of these features can impact the swimmer–swimmer hydrodynamic interactions, especially in semi-dilute to dense suspensions. As we will see in the following sections, these features, linked to the dynamics at the level of individual cells, do indeed propagate to larger scales and can significantly affect the observed coherent structures and the processes by which they form.

## 4. Suspensions with a monodisperse distribution of undulation frequencies form clusters

Using the methods described in the previous section, we simulate the dynamics of a suspension of *N* = 1000 swimmers with *N*_flag_ = 29 for 200 undulation periods. In this simulation, all swimmers are driven by a prescribed preferred curvature, equation (2.1), with the same undulation frequency, *ω* = *K*_B_(Sp/*l*)^4^/(4*πη*), and wavenumber *k* = 2*π*/*l*. Each swimmer has a different phase, *φ*, drawn randomly from a uniform distribution. The swimmers are initially distributed uniformly in the domain and are aligned to swim in the −*x*-direction. Swimmer–swimmer hydrodynamic interactions are incorporated by solving the full mobility problem that couples the motion of all swimmer segments and heads, while steric interactions are included through short-ranged, pairwise, repulsive forces between all flagellum segments and cell heads. The effective area fraction is *ν* = *Nd*^2^/(4*L*^2^) = 1.42. Based on the film thickness, the effective volume fraction, as used in [22], is approximately 2.56. For suspensions of pusher dipoles at this effective volume fraction, one would expect rapid decay of the initial polar order followed by the onset of large-scale, fluctuating motion [19,22].

**Figure 3.**
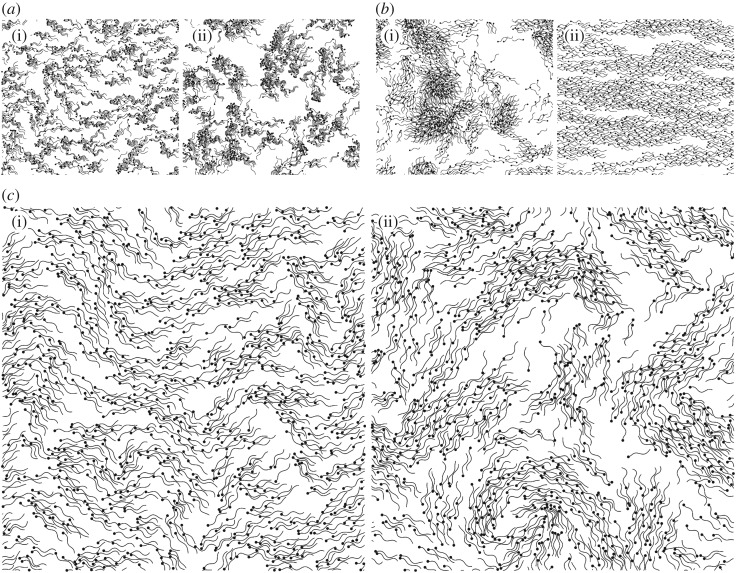
Evolution of suspensions of 1000 swimmers. (*a*) Snapshots of a system with fixed undulation frequency taken after 30 (i) and 198 (ii) periods. (*b*) Snapshots after 340 undulation periods from two stochastically varying frequency RFT simulations starting from isotropic (i) and polar (ii) initial configurations. (*c*) Snapshots of a simulation with randomly varying frequencies starting from a polar configuration at 60 (i) and 198 (ii) undulations.

Figure 3*a* shows the suspension after 30 and 198 undulation periods. The corresponding video showing the complete evolution is provided as electronic supplementary material. While the suspension quickly evolves from the initial polar state, the dynamics are very different from the long-wavelength bending instabilities predicted by both simulations of rigid, steady pushers [18,22] and kinetic or continuum models [17,19–21,24]. Instead, we find that the swimmers have a strong tendency to aggregate and form clusters within which flagella are aligned. A quantitative analysis of this cluster growth is included in the electronic supplementary material. Once small clusters have formed, they remain intact and can attract other clusters in their vicinity. As two attracting clusters approach each other, they either rotate to swim in the same direction and merge to form a bigger, synchronized cluster, or instead swim in opposing directions and move apart at an enhanced relative velocity. Earlier simulations of sperm [28,29] showed similar aggregation dynamics in two-dimensional suspensions, but here we see that aggregation also dominates in larger, denser suspensions where long-range hydrodynamics could be expected to produce large-scale swirls and jets.

**Figure 4.**
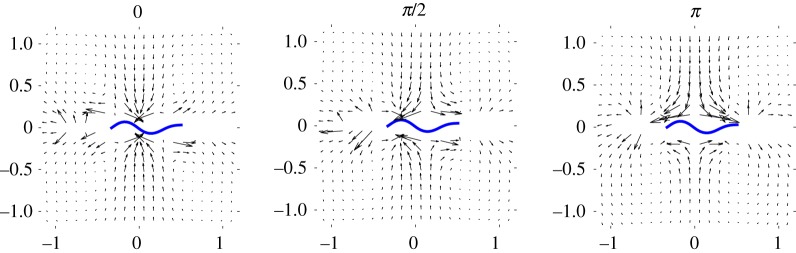
Relative centre of mass displacements (in units of swimmer length) over four periods of undulation for two parallel swimmers with relative phase shifts Δ*φ* = 0, *π*/2 and *π*. For clarity, the cell heads attached to the left ends of the flagella are omitted. (Online version in colour.)

The aggregation process is driven by the hydrodynamic attraction and phase-locking behaviour that has been found previously with models of undulatory swimmers [26–29,34,58]. We measure this for our system by examining in detail the pairwise interactions between two swimmers moving in the −*x*-direction with phase differences Δ*φ* = 0, *π*/2 and *π*. The vector fields in figure 4 show the displacement over four undulation periods of one swimmer centred at (*x*, *y*) relative to another placed at the origin. When the swimmers are far apart, all three vector fields resemble the displacement field associated with pusher dipoles. At smaller separations, however, the fields differ and the influence of the phase difference can be readily seen. We see that there is clear attraction, if the swimmers are in phase. When the phase difference is *π*/2, the swimmers attract and simultaneously move relative to each other as to align the crests of the flagellar waves. If they are out of phase, the swimmers again move relative to one another attempting to align wave crests; however, the relative shift is much larger. The large displacements observed near the swimmer's ends are due to the steric forces between the head of one swimmer and the flagellum of the other.

## 5. Suspensions with stochastically varying frequencies reach a turbulence-like state

In the previous section, we saw that flagellum undulations can produce flows that lead to hydrodynamically induced aggregation and cluster formation. While this provides a mechanism for the formation of coherent groups, such as wood mouse sperm trains [11], the patterns differ markedly from the large-scale swirling found in experiments on ram sperm suspensions [14,15]. In real samples, sperm cells have distributions of waveforms, undulation frequencies or geometries. Accordingly, the parameters appearing in our model should not necessarily be the same for all individuals and should be chosen to accurately describe variations from individual to individual across the population.

We explore how variations in individual dynamics across the population impact large-scale suspension dynamics by introducing stochastic modulations of the undulation frequency into the model, allowing flagellar waves to differ from individual to individual and to vary over time [46,59]. As we solve for the flagellum dynamics, changing the frequency also leads to changes in the waveform. The individual frequencies are drawn from a lognormal distribution after time intervals of length *T* = 2*π*/*ω* as described in the electronic supplementary material. We set the mean frequency to *ω* and, in view of matching experimental data [45,59], set the standard deviation *σ*_*ω*_ to be *ω*/5.

We repeat our simulations of 1000 swimmers, but now allowing for stochastic variations in the frequencies. Snapshots of the suspension evolution are shown in figure 3*c* and corresponding videos can be found as part of the electronic supplementary material. The stochastic variations in the waveforms suppress the ability of neighbouring flagella to synchronize. Rather than forming clusters, the swimmers now form waves, swirls and vortices, as seen in concentrated, quasi-two-dimensional ram sperm suspensions [14]. By running an equivalent simulation starting from an isotropic initial configuration, it was verified that the qualitative features of the fully nonlinear state are independent of the initial conditions. We also note that a sufficiently large *σ*_*ω*_ is needed to successfully suppress aggregation and observe large-scale motion of the suspension. Simulations of smaller ensembles (see the electronic supplementary material) show that lower values of *σ*_*ω*_ still exhibit cluster formation (e.g. *σ*_*ω*_ = *ω*/20 or *σ*_*ω*_ = *ω*/50). In these cases, stochastic frequency changes are rarely large enough to break the synchronization of adjacent flagella.

While hydrodynamic interactions no longer lead to aggregation, they still play a strong role in the evolution of the suspension. We perform simulations where we have removed the hydrodynamic interactions by solving the mobility problem using resistive force theory (RFT) instead of FCM. As described in the electronic supplementary material, the drag coefficients appearing in the RFT are chosen as to closely match the swimming speeds and flagellar waves obtained with the full FCM model. Figure 3*b* shows snapshots from two different RFT simulations with *ν* = 1.42 and *N* = 1000 where the initial swimmer directions are either all aligned or distributed isotropically. The corresponding videos are provided as part of the electronic supplementary material. The time-evolution of these suspensions is very different from those with hydrodynamic interactions. Most strikingly, for the initially polar suspension, long bending-waves are entirely absent and at long times we observe ‘laning’ states similar to those found in simulations of self-propelled rods without hydrodynamic interactions [9,29,60]. When the initial state is isotropic, laning is not observed (figure 3*b*), indicating further differences between the RFT and full simulations. Additionally, in thin films, the hydrodynamic interactions between the swimmers are longer ranged than they would be in bulk. As a result, we find that as we increase the film thickness, though the instability of the polar state takes longer to develop, we still observe (see the electronic supplementary material) the eventual onset of large-scale motion qualitatively similar to that found in the thinner film cases.

## 6. Order parameters exhibit density dependence

The polar and nematic order of the suspension is dependent on the effective area fraction, *ν*. We perform a series of smaller stochastic simulations (*σ*_*ω*_ = *ω*/5) ranging from 170 to 500 swimmers run to *t* = 400 *T* and compare the order parameters6.1
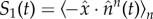
and6.2
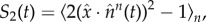
which measure the global alignment with the initial swimming direction (*S*_1_) and the global nematic order with respect to the initial direction (*S*_2_) [22]. Here, 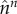 is the *n*th swimmer's mean orientation and 〈 · 〉_*n*_ denotes the average over all swimmers. Videos of the simulations are provided as the electronic supplementary material.

**Figure 5.**
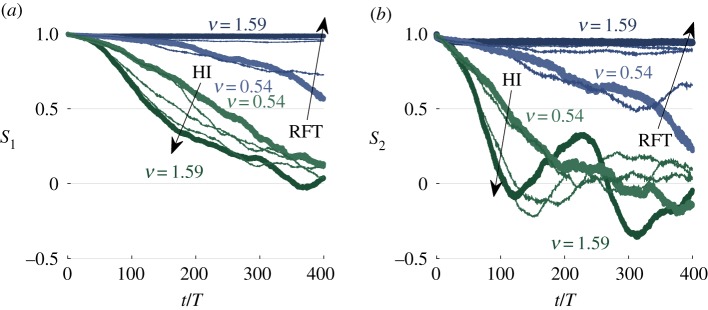
Evolution of the global order parameters *S*_1_ (*a*) and *S*_2_ (*b*) over 400 periods for initially aligned suspensions with *σ*_*ω*_ = *ω*/5, *L* = 8.86*d* and *ν* = 0.54–1.59. Simulations are performed with full hydrodynamic interactions (HI, green) and with steric interactions only (RFT, blue), which show the opposite trend with *ν*. (Online version in colour.)

We find that the directional order, given by *S*_1_, decreases with time and the decay rate increases with swimmer density (figure 5*a*). The time scale of the decay is comparable to that found for rod models [22,23]. The nematic order, given by *S*_2_, initially decreases with time. However, for higher densities, we see large fluctuations emerge after the initial decay (figure 5*b*). The appearance of these fluctuations during the relatively gentle decay of *S*_1_ reflects periodic folding and rotation of large patches of aligned swimmers. Figure 5 also shows *S*_1_ and *S*_2_ for initially polar RFT simulations with the same effective area fractions. With hydrodynamic interactions removed, we find that higher density suspensions retain their alignment for longer times due to enhanced local caging.

## 7. Flagellar undulations contribute significantly to the energy and velocity spectra

**Figure 6.**
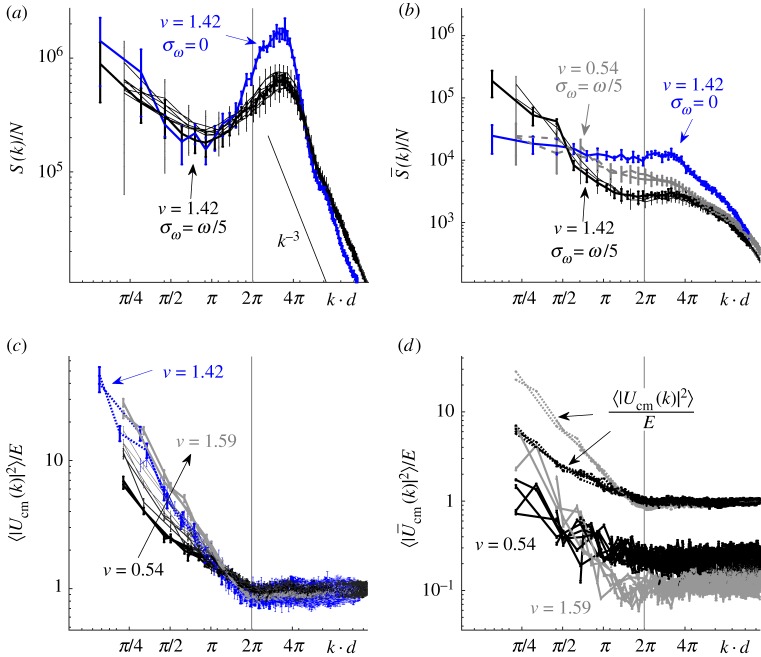
Fluid energy and centre of mass velocity spectra for suspensions with effective area fractions *ν* = 0.54–1.59 in domains of size *L* = 8.86*d* and *L* = 13.29*d* with both fixed (*σ*_*ω*_ = 0) and stochastically (*σ*_*ω*_ = *ω*/5) varying frequencies. For the cases where *L* = 8.86*d* the averaging is performed over times between 200*T* and 400*T*, while for *L* = 13.29*d*, the interval is from 150*T* to 200*T*. (*a*) Fluid energy spectrum *S*(*k*)/*N*. The error bars indicate the sample standard deviation. (*b*) Fluid energy spectrum 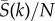 of the filtered velocity field from which the short time scales have been removed. (*c*) Centre of mass velocity spectrum for all stochastic simulations. The data are normalized by the total energy, 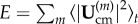, for each case. The error bars indicate one standard error of the mean. (*d*) Centre of mass velocity spectrum of the filtered centre of mass velocity fields for the lowest and highest *ν*. The spectra for the respective unfiltered fields from (*c*) are included for comparison. (Online version in colour.)

To further quantify the large-scale motion, we compute the fluid energy spectrum,7.1
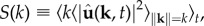
where 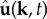 is the spatial Fourier transform of the fluid velocity and 〈 · 〉_*t*_ denotes time-averaging while 〈 · 〉_∥**k**∥=*k*_ signifies averaging over all wave vectors of magnitude *k*. Figure 6*a* shows *S*(*k*)/*N* from simulations with both fixed and stochastically varying frequencies and for different values of *ν*. With the exception of the pronounced peak near the undulation wavelength, the spectra are similar to those found in experiments on concentrated sperm suspensions [14]. In particular, we also find that the spectra decay like *k*^−3^ for large *k* (figure 6*a*). Given that this power-law decay appears over sub-swimmer length scales, we associate it with random forcing of the fluid by the flagella. A calculation presented in the electronic supplementary material demonstrates that an identical decay is obtained when the fluid is driven by spatially uncorrelated forcing. This is different from the *k*^−4^ decay observed at small scales in suspensions of self-propelled rods [23]. This decay was attributed to the sharp jump in the forcing along the length of each swimmer. Thus, the details regarding how the swimmers propel themselves can lead to changes in the spectrum at length scales comparable to the swimmer size.

Further, we find that the fluid energy spectrum is dominated by contributions at the time scale of flagellar undulations, even at long length scales. Despite qualitative differences in their dynamics and the quantitative differences in the evolution of the order parameters, *S*(*k*)/*N* is nearly identical for all stochastic simulations, regardless of *ν*. The collapse illustrates that *S*(*k*) scales with the number of swimmers. This is in contrast with large increases with *ν* seen at low *k* in simulations of self-propelled rod suspensions [23]. Surprisingly, the *S*(*k*)/*N* curves for the fixed and stochastically varying frequency simulations also do not differ drastically, despite the striking differences in their suspension evolution. We expect that this is due to the energy injected at the short time scales associated with flagellar undulations being large compared with that due to the large-scale motion of the suspension. To limit the contribution of short time scales to *S*(*k*), we apply a low-pass filter to 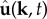 by computing a running time-average of **u**(***x***, *t*) with a window of 8*T* before performing the transform. The spectra, 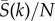, computed using the filtered velocity fields are shown in figure 6*b*. With the short time scales suppressed, the dependence on *ν* becomes visible. We now observe larger values of 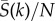 at low *k* when the sperm density is high, revealing quantitatively the large-scale fluid motion at the time scale of suspension evolution. Additionally, the spectrum for the fixed-frequency simulations is now almost flat at small *k* indicative of the lack of long-range correlations.

Following from previous work on bacterial suspensions [9,60], we also examine the spectrum of the centre of mass velocity field, 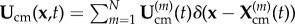, where ***X***^(*m*)^_cm_ and **U**^(*m*)^_cm_ are the centre of mass position and velocity, respectively, as defined in the electronic supplementary material. Figure 6*c* shows the swimmer velocity spectrum, 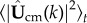, for different values of *ν*. For each *ν*, the lowest spatial modes (lowest *k*) provide the largest contribution to the total spectrum, with the contribution increasing as *ν* increases. From these maximum values, the spectra then quickly decrease with *k* to a flat profile at scales below the flagellum length, corresponding to the level of noise generated in constructing the swimmer velocity field.

Due to flagellar undulations, the swimmers' centre of mass velocities oscillate about their mean values at the time scale of the undulation frequency. We can again remove contributions of the short time scale by filtering the swimmers' centre of mass velocities using a running time-average with window size 8*T*. Figure 6*d* shows the spectra 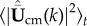 associated with the filtered swimmer velocity field. Filtering reduces values of the swimmer velocity spectrum for all *k*, indicating contributions at short time scales at all length scales. For lower values of *ν*, the spectrum is reduced by an approximately constant factor across all *k*, while for higher values of *ν*, the reduction is more pronounced at shorter scales.

## 8. Discussion and conclusions

In this study, we have used numerical simulation to show that variability in sperm flagellum dynamics across a suspension can lead to large changes in collective dynamics. We have incorporated these variations through stochastic changes in the actuation frequency that, in turn, lead to changes in waveform and amplitude. In actual sperm suspensions, variations are also likely to be present in flagellum length and cell geometry, and further, the flagellar waveform is likely to be more complex and fully three-dimensional [47,61]. We expect these features to have a similar, aggregation-limiting effect as the stochastic variation in frequency. As such, measurements quantifying the distributions of individual sperm properties could not only provide a better classification of the individual cells, but also a better understanding of their collective dynamics.

Our fixed frequency simulations show that flagellar synchronization leads to aggregation and clustering. Certain properties associated with sperm that are found to self-organize into coherent groups, such as wood mouse sperm, might promote synchronization. It is known that these sperm typically have flagella that are longer than those of sperm from other species. By allowing for larger amplitudes, longer flagella may enhance the higher-order, time-dependent flows that we find are responsible for aggregation. Longer, thinner flagella would also bend and flex with greater ease in response to flows generated by neighbouring sperm, further reinforcing the tendency to synchronize and aggregate. Additionally, microtubule sliding driving the undulations may depend on the external load experienced by the flagellum. This could allow for a non-trivial coupling between the actuation mechanism and the surrounding flow field, which, in turn, may also promote synchronization of neighbouring flagella and cluster formation.

The turbulence-like state achieved when there is sufficient variation in the undulation frequency is both quantitatively and qualitatively similar to that observed in suspensions of ram and bull semen. While longer time simulations would be needed to explore this state in more detail, simulations starting from either polar or isotropic states eventually exhibit similar qualitative dynamics and have the same fluid energy spectrum at the final simulation times. Both this and the many previous results obtained using reduced models suggest that the observed jets and swirls are features of a final turbulence-like state. The presence of this state has been empirically correlated with sample fertility [62]. We have shown that its onset depends on sperm density, and its observation could therefore be serving as an indicator of sperm count, a well-known measure of sample fertility.

While in the simulations presented here the fluid domain is fully three-dimensional, the motion of the sperm cells is restricted to a plane. Even though layering in sperm suspensions has been observed in experiments [14] and sperm are attracted to move along planar surfaces [63], the imposed two-dimensional motion in our simulations may enhance the role of steric interactions. For example, in a real sample when two sperm cells collide, it is likely that one will pass over the other and they will both experience some change in their swimming directions. In our simulations, the sperm cannot pass by each other as easily and the collisions can dramatically affect their swimming directions, leading to near alignment or anti-alignment. The interactions of self-driven filaments [58] and the trajectories of interacting sperm pairs [34] have recently been explored in simulation. These studies have shown that perturbations from planarity can lead to more complex trajectories, as well as a dependence on the elasticity of the flagella. With further advances in numerical methods for simulating flexible filaments and more accurate models for three-dimensional flagellar waveforms, it will be possible to explore these effects through direct simulation of sperm suspensions. Our theoretical model for the elastic flagellum and the FCM approach to the mobility problem carry over to fully three-dimensional simulations. While there will be additional computational costs associated with the increased size of the fluid domain, more limiting is the technical challenge involved in keeping track of the fully three-dimensional deformation of the flagellum and, at the same time, using implicit time integration to handle numerical stiffness. We are currently developing the numerical methods to address this challenge.

Additionally, it would be of interest to ascertain suspension dynamics at even larger length scales and for longer time scales than those that may be accessible through direct simulation. Continuum models incorporating the time dependence of the flows generated by the swimmers have recently been developed [64–66] and could possibly be adapted to capture the effects of flagellar undulations seen in our simulations and used to explore nonlinear suspension dynamics at these scales.

Along with variations among the cells, collective dynamics are likely to be influenced by more complex features of the environment, such as nearby boundaries that could curve sperm trajectories [59], or particles or other microscale structures in the surrounding fluid that are known to enhance swimming speeds of undulatory swimmers [35]. Non-Newtonian features of the surrounding fluid, such as viscoelasticity, typically associated with biological fluids have also been found to promote clustering [67]. These, and other important, perhaps more complex effects, such as chemotaxis, could further contribute to the richness and diversity of sperm collective dynamics.

## Supplementary Material

Supporting Information

## Supplementary Material

Flagellar undulations in a suspension synchronized sperm

## Supplementary Material

Aggregation in a synchronized sperm suspension

## Supplementary Material

Large-scale motion in a sperm suspension

## Supplementary Material

Evolution of an initially isotropic sperm suspension in the absence of hydrodynamics interactions

## Supplementary Material

Evolution of an initially polar sperm suspension in the absence of hydrodynamics interactions

## Supplementary Material

Dynamics of an initially isotropic low-density sperm suspension

## Supplementary Material

Dynamics of an initially isotropic high-density sperm suspension

## Data accessibility

This article has no additional data.
