# Supplementary material for: From flagellar undulations to collective motion: predicting the dynamics of sperm suspensions
Source: J R Soc Interface. 2018 Mar 21;15(140):20170834. doi: 10.1098/rsif.2017.0834 (PMC5908526; doi:10.1098/rsif.2017.0834)
Supplement: Supporting Information [file rsif20170834supp1.pdf]

From flagellar undulations to collective motion: predicting the dynamics of sperm suspensions

Simon F. Schoeller and Eric E. Keaveny*

Department of Mathematics
Imperial College London
South Kensington Campus
London SW7 2AZ, UK

February 25, 2018

Supporting Information

Swimmer model

As each swimmer is identical, for clarity, we describe the model for a single swimmer and subsequently describe how it generalizes when considering a suspension. Recall that a swimmer is composed of two elements, its cell head and its flagellum, where the cell head is treated as a rigid oblate spheroid with semi-major and minor axes a and b, respectively. Using the arc-length parametrization where $\hat{\boldsymbol{t}}(s) = d\mathbf{Y}/ds$, the force and moment balance equations are

$$\frac{d\boldsymbol{\Lambda}}{ds} + \boldsymbol{f} = 0$$
$$\frac{d\boldsymbol{M}}{ds} + \boldsymbol{\tau}^D + \hat{\boldsymbol{t}} \times \boldsymbol{\Lambda} + \boldsymbol{\tau} = 0.$$

where $\boldsymbol{\Lambda}$ is the tension and $\boldsymbol{M} = K_B \hat{\boldsymbol{t}} \times d\hat{\boldsymbol{t}}/ds$ is the bending moment. The external force per unit length, $\boldsymbol{f}$, and torque per unit length, $\boldsymbol{\tau}$, arise due to the viscous stresses along the flagellum and, in the case of multiple swimmers, steric interactions between neighboring swimmers. The torques $\boldsymbol{\tau}^D$ per unit length arise due to the time-dependent preferred curvature. At the free end $(s = l)$, the boundary conditions are such that the tension and moment are zero, while at the attachment point to the cell head, we require a clamped-end condition be satisfied.

To solve these equations numerically, we first discretize the flagellum into N_{flag} segments of length ΔL. The segments have positions $\boldsymbol{Y}_n$ and orientations $\hat{\boldsymbol{t}}_n$ for $n = 1, \ldots, N_{\text{flag}}$. The orientations are the discrete representation of the centerline tangents at the segment positions. Considering the tension and bending moment at the midpoints between adjacent segments and applying central differencing, we obtain the discretized force and torque balances

$$\frac{\boldsymbol{\Lambda}_{n+1/2} - \boldsymbol{\Lambda}_{n-1/2}}{\Delta L} + \boldsymbol{f}_n = 0 \tag{1}$$
$$\frac{\boldsymbol{M}_{n+1/2} - \boldsymbol{M}_{n-1/2}}{\Delta L} + \frac{1}{2}\hat{\boldsymbol{t}}_n \times (\boldsymbol{\Lambda}_{n+1/2} + \boldsymbol{\Lambda}_{n-1/2}) + \boldsymbol{\tau}_n^D + \boldsymbol{\tau}_n = 0, \tag{2}$$

*e.keaveny@imperial.ac.uk

where $\boldsymbol{M}_{n+1/2} = (K_B/\Delta L)\hat{\boldsymbol{t}}_n \times \hat{\boldsymbol{t}}_{n+1}$. Multiplying through by ΔL and introducing $\boldsymbol{F}_n = \boldsymbol{f}_n \Delta L$ and $\boldsymbol{T}_n = \boldsymbol{\tau}_n \Delta L$ as the total applied force and torque, respectively, on segment n, we may write Eqs. [1] and [2] as

$$\boldsymbol{F}_n^C + \boldsymbol{F}_n = 0, \tag{3}$$

$$\boldsymbol{T}_n^B + \boldsymbol{T}_n^C + \boldsymbol{T}_n^D + \boldsymbol{T}_n = 0, \tag{4}$$

where $\boldsymbol{F}_n^C = \boldsymbol{\Lambda}_{n+1/2} - \boldsymbol{\Lambda}_{n-1/2}$, $\boldsymbol{T}_n^B = \boldsymbol{M}_{n+1/2} - \boldsymbol{M}_{n-1/2}$, and $\boldsymbol{T}_n^C = (\Delta L/2)\hat{\boldsymbol{t}}_n \times (\boldsymbol{\Lambda}_{n+1/2} + \boldsymbol{\Lambda}_{n-1/2})$. The driving torques, $\boldsymbol{T}_n^D$, arising from the preferred curvature are given by

$$\boldsymbol{T}_n^D = K_B(\kappa(s_n, t) - \kappa(s_{n+1}, t))\hat{z}$$

where $s_n = (n - 1/2)\Delta L$ for $n = 1, \ldots, N_{\text{flag}}$, and

$$\kappa(s,t) = K_0 \sin(ks - \omega t + \varphi) \cdot \begin{cases} 2(l-s)/l, & s > l/2 \\ 1, & s \leq l/2, \end{cases}$$

as well as, $\kappa(s_1, t) = \kappa(s_{N_{\text{flag}}+1}, t) = 0$.

The tension $\boldsymbol{\Lambda}_{n+1/2}$ enforces flagellum inextensibility at the level of each segment. In the discrete setting, they are the Lagrange multipliers associated with the constraints,

$$\boldsymbol{Y}_{n+1} - \boldsymbol{Y}_n + \frac{\Delta L}{2}(\hat{\boldsymbol{t}}_n + \hat{\boldsymbol{t}}_{n+1}) = 0. \tag{5}$$

In the discretized system, the boundary conditions at the free end are satisfied by taking $\boldsymbol{\Lambda}_{N_{\text{flag}}+1/2} = \boldsymbol{M}_{N_{\text{flag}}+1/2} = 0$. The clamped-end condition on the cell head is recovered by treating the head as another segment of size $2a$ with the bending moment and inextensibility constraint computed with respect to the attachment point rather than the head center.

Until now, we have discussed the model in the context of a single swimmer. To allow for N swimmers, we must have N force and moment balances, one pair for each of the flagella. As the driving torques, tension, and bending moments are internal to each flagellum, the force and moment balances are coupled only through the external forces and torques. In our simulations, the coupling arises due to steric repulsion between the segments and heads and hydrodynamic interactions. Accordingly, the total external force on segment n is given by $\boldsymbol{F}_n = \boldsymbol{F}_n^S + \boldsymbol{F}_n^H$ where $\boldsymbol{F}_n^S$ is the steric force and $\boldsymbol{F}_n^H$ is the hydrodynamic force on the segment. The only external torque on the segment is the viscous torque, $\boldsymbol{T}_n = \boldsymbol{T}_n^H$

The steric force $\boldsymbol{F}_n^S$ on segment n is given by the generic repulsive barrier force

$$\boldsymbol{F}_n^S = -F_S \sum_{m \in I(n)} \left(\frac{(\chi R_{nm})^2 - |\boldsymbol{Y}_n - \boldsymbol{Y}_m|^2}{(\chi R_{nm})^2 - R_{nm}^2} \right)^4 \frac{(\boldsymbol{Y}_n - \boldsymbol{Y}_m)}{d},$$

where the sum runs over $I(n)$, the set of all segments/heads, m, with $|\boldsymbol{Y}_n - \boldsymbol{Y}_m| < \chi R_{nm}$ and $\chi = 1.1$. The parameter R_{nm} specifies the center-to-center distance for particles n and m at contact and χ sets the range over which the barrier force acts. If n and m are both segments, the contact distance is $R_{nm} = 2b$, where as if n and m are both heads, we have $R_{nm} = 2a$. Lastly, if n and m are a head-segment pair then $R_{nm} = a + b$. The parameter F_S sets the strength of the repulsion at contact, which in our simulations is $F_S = 9018 K_B/d^2$.

Force-coupling method

The force and torque balances, Eqs. (3) and (4) respectively, establish a low Reynolds number mobility problem whose solution is the coupled motion of the particles through the fluid. To solve this mobility problem, we utilize the force-coupling method (FCM) [7, 6, 5], which, for the sake of clarity, we describe here in the case of spherical particles of equal radius, however, it readily extends to spheroidal particles [5], such as our cell heads, and allows for polydispersity in particle sizes.

In FCM forces and torques on the particles are projected onto the fluid using a truncated and regularized force multipole expansion. This leads to the incompressible Stokes flow

$$\eta\nabla^2 \boldsymbol{u} - \boldsymbol{\nabla} p = \sum_n \boldsymbol{F}_n^H \Delta_n(\boldsymbol{x}) - \frac{1}{2}\sum_n \boldsymbol{T}_n^H \times \boldsymbol{\nabla}\Theta_n(\boldsymbol{x})$$
$$\boldsymbol{\nabla}\cdot\boldsymbol{u} = 0,$$

for pressure field, p, and fluid velocity field, $\boldsymbol{u}$. The Stokes flow is generated by the forces and torques each particle n exerts on the fluid, which, in the case of our swimmers, will be $\boldsymbol{F}_n^H = -\boldsymbol{F}_n^C - \boldsymbol{F}_n^S$, and $\boldsymbol{T}_n^H = -\boldsymbol{T}_n^B - \boldsymbol{T}_n^C - \boldsymbol{T}_n^D$. The force and torques are projected to the fluid via the Gaussian functions, which for particle n are given by

$$\Delta_n(\boldsymbol{x}) = \left(2\pi\sigma_\Delta^2\right)^{-3/2} \exp\left[-\left|\boldsymbol{x} - \boldsymbol{Y}_n\right|^2/(2\sigma_\Delta^2)\right],$$
$$\Theta_n(\boldsymbol{x}) = \left(2\pi\sigma_\Theta^2\right)^{-3/2} \exp\left[-\left|\boldsymbol{x} - \boldsymbol{Y}_n\right|^2/(2\sigma_\Theta^2)\right].$$

The motion of the particles is obtained by volume averaging the resulting fluid velocity using the same Gaussian functions. Specifically, the translational and angular velocities are given by

$$\boldsymbol{V}_n = \int \boldsymbol{u}\,\Delta_n(\boldsymbol{x})\,d^3\boldsymbol{x}$$
$$\boldsymbol{\Omega}_n = \frac{1}{2}\int (\boldsymbol{\nabla}\times\boldsymbol{u})\,\Theta_n(\boldsymbol{x})\,d^3\boldsymbol{x}.$$

If the particles have radius A, FCM reproduces the correct Stokes drag and viscous torque with $\sigma_\Delta = A/\sqrt{\pi}$ and $\sigma_\Theta = A/(6\sqrt{\pi})^{1/3}$. In our simulations, we preserve these ratios between the particle and the Gaussian envelope sizes. The segments are taken to have radius $b = \Delta L/2.2$, while the semi major-axes of the spheroidal heads are $a = 3b$ and the semi minor-axes are b. All simulation parameters are summarized in table 1 and the datasets provided have the same units.

Updating positions and orientations

As our simulations are quasi-2D in that the motion of the swimmers is restricted to a plane, we have $\boldsymbol{\Omega}_n = \Omega_n\hat{\boldsymbol{z}}$ for all n and can introduce angle θ_n such that $\hat{\boldsymbol{t}}_n = (\cos\theta_n, \sin\theta_n)$. Therefore, after computing the $\boldsymbol{V}_n$ and $\boldsymbol{\Omega}_n$ for each segment and head, the positions and orientations are obtained by integrating in time

$$\frac{d\boldsymbol{Y}_n}{dt} = \boldsymbol{V}_n \quad (6)$$
$$\frac{d\theta_n}{dt} = \Omega_n. \quad (7)$$

subject to the inextensibility constraints given by [5]. To do this numerically, we rely on a second-order implicit BDF scheme [1] and Broyden's method [2] to find the updated positions and orientations, as well as the Lagrange multipliers $\boldsymbol{\Lambda}_{n+1/2}$.

Swimmer center of mass and director

We define the center of mass of the m-th swimmer as

$$\boldsymbol{X}_{\text{cm}}^{(m)} = \frac{1}{a^2 + N_{\text{flag}}b^2}\left(a^2\boldsymbol{Y}_0^{(m)} + b^2\sum_{n=1}^{N_{\text{flag}}}\boldsymbol{Y}_n^{(m)}\right).$$

Here, $\boldsymbol{Y}_0^{(m)}$ is the position of the m-th swimmer's head, and $\boldsymbol{Y}_k^{(m)}$ is that of its k-the segment. The center of mass velocity is then given by

$$\boldsymbol{U}_{\text{cm}}^{(m)} = \frac{1}{a^2 + N_{\text{flag}}b^2}\left(a^2\boldsymbol{V}_0^{(m)} + b^2\sum_{n=1}^{N_{\text{flag}}}\boldsymbol{V}_n^{(m)}\right).$$

The director of swimmer m is given by

$$\hat{n}^{(m)} = -\frac{1}{N_{\text{flag}}+1}\sum_{n=0}^{N_{\text{flag}}} \hat{\boldsymbol{t}}_n^{(m)}.$$

Force multipole expansion for a single swimmer

The singular force density for a single swimmer is given by

$$f_i(\boldsymbol{x}) = \sum_n F_i^n \delta(\boldsymbol{x}-\boldsymbol{Y}_n) + \sum_n G_{ij}^n \partial_j \delta(\boldsymbol{x}-\boldsymbol{Y}_n),$$

where the asymmetric dipole is related to the torque through $G_{ij} = \epsilon_{ijk}T_k/2$. We have suppressed the superscript index, since we are considering only one swimmer. Expanding this force density about the swimmer's center of mass, $\boldsymbol{X}_{\text{cm}}$, and defining the position of the center of mass relative to the n-th segment/head as $\boldsymbol{R}^n = \boldsymbol{X}_{\text{cm}} - \boldsymbol{Y}_n$, we have

$$\begin{aligned} f_i(\boldsymbol{x}) = & \\ = & \underbrace{\left(\sum_n F_i^n\right)}_{=0} \delta(\boldsymbol{x}-\boldsymbol{X}_{\text{cm}}) + \\ + & \left(\sum_n G_{ij}^n + F_i^n R_j^n\right) \partial_j \delta(\boldsymbol{x}-\boldsymbol{X}_{\text{cm}}) + \\ + & \left(\sum_n G_{ij}^n R_k^n + \frac{1}{2} F_i^n R_j^n R_k^n\right) \partial_k \partial_j \delta(\boldsymbol{x}-\boldsymbol{X}_{\text{cm}}) + \\ + & \text{ higher order terms.} \end{aligned}$$

The first term is zero because there is no external force on the swimmer. The remaining two terms are the force dipole and quadrupole,

$$\begin{aligned} G_{ij} &\equiv \sum_n G_{ij}^n + F_i^n R_j^n \\ K_{ijk} &\equiv \sum_n G_{ij}^n R_k^n + \frac{1}{2} F_i^n R_j^n R_k^n, \end{aligned}$$

respectively, which drive the flow observed in the limit $|\boldsymbol{x} - \boldsymbol{X}_{\text{cm}}| \gg d$. We compute the symmetric, traceless and anti-symmetric parts of these tensors, given by

$$\begin{aligned} G_{ij}^S &= \frac{1}{2}(G_{ij} + G_{ji}) - \frac{1}{3} G_{kk}\delta_{ij} \\ G_{ij}^A &= \frac{1}{2}(G_{ij} - G_{ji}) \\ K_{ijk}^S &= \frac{1}{2}(K_{ijk} + K_{jik}) - \frac{1}{3} K_{mmk}\delta_{ij} \\ K_{ijk}^A &= \frac{1}{2}(K_{ijk} - K_{jik}) \end{aligned}$$

as functions of time. These are shown in Fig. 2 in the main text, with the exception of $G^A(t)$ which is zero for all time as there is no net-torque acting on the swimmers.

Stochastic variation of the undulation frequency

We introduce stochastic changes of the undulation frequencies to explore how variability within a population, as well as the fluctuations of individual frequencies over time can impact collective dynamics. After every

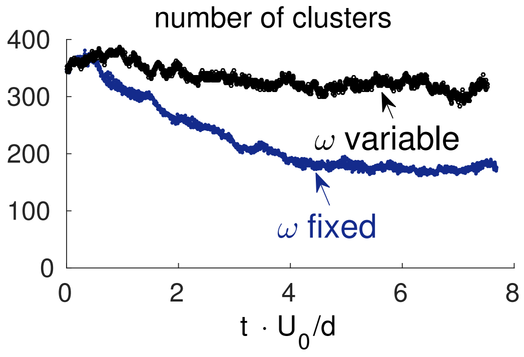

Figure 1: Number of clusters over time in simulations of 1000 swimmers starting from a polar configuration. Time is normalized by the average swimming velocity U_0 and the swimmer length d.

fixed average period $T = 2\pi/\omega$, a new undulation frequency, ω_B, is randomly assigned to a swimmer to replace its previous value, ω_A. New frequencies are drawn from a log-normal distribution with parameters $(\mu_{\text{ln}}, \sigma_{\text{ln}})$ given by

$$\mu_{\text{ln}} = \log\left(\frac{\omega^2}{\sqrt{\omega^2 + \sigma_\omega^2}}\right)$$
$$\sigma_{\text{ln}} = \sqrt{\log\left(\delta_\omega^2 + 1\right)},$$

where the mean frequency is ω and the standard deviation of the distribution is $\sigma_\omega = \delta_\omega \omega$. To enforce continuity in time for each swimmer's preferred curvature $\kappa(s,t)$, the phase must also be updated from φ_A to φ_B. Specifically, at the time t_{AB} when the frequency changes, we require

$$ks - \omega_A t_{AB} + \varphi_A - (ks - \omega_B t_{AB} + \varphi_B) \in 2\pi\mathbb{Z}.$$

This can be fulfilled by taking

$$\varphi_B = 2\pi\left(\frac{(\omega_B - \omega_A)t_{AB} + \varphi_A}{2\pi} - \left\lfloor \frac{(\omega_B - \omega_A)t_{AB} + \varphi_A}{2\pi} \right\rfloor\right),$$

for each individual swimmer.

We note that an alternative approach to introducing stochasticity is to assign a random frequency to each swimmer at the beginning of the simulation and keep it fixed for all time. This was adopted previously in [8]. We found that on time-scales that are relatively short compared to the evolution of the suspension this leads to qualitatively similar results as those obtained by allowing the frequency to vary over time. Over longer times, however, we expect, based on our monodisperse frequency simulations, that keeping the frequencies fixed could potentially lead to preferential clustering of swimmers with similar frequencies and hence structurally different dynamics.

Identifying and counting swimmer clusters

To effectively identify swimmer clusters, we must account for relative swimmer orientations in addition to relative distances. Based on this, swimmer i is taken to belong to a cluster if the modified distance

$$d^\beta(i,j) \equiv \left|\boldsymbol{X}_{\text{cm}}^{(i)} - \boldsymbol{X}_{\text{cm}}^{(j)}\right| + \beta \tan\left(\frac{1}{2}\arccos\left(\hat{n}^{(i)} \cdot \hat{n}^{(j)}\right)\right)$$
$$= \left|\boldsymbol{X}_{\text{cm}}^{(i)} - \boldsymbol{X}_{\text{cm}}^{(j)}\right| + \beta\left(\frac{\sqrt{1 - \hat{n}^{(i)} \cdot \hat{n}^{(j)})}}{\sqrt{1 + \hat{n}^{(i)} \cdot \hat{n}^{(j)}}}\right)$$

with at least one other swimmer j in the cluster is $d^\beta(i,j) < d/3$. The parameter β controls the influence of particle alignment and is chosen to be $\beta/d = \frac{1}{6}\frac{1}{\tan(\pi/8)} = (\sqrt{2}-1)/6$. With this choice of β, swimmers with orientations differing by an angle of $\pi/4$ must be half as far apart as aligned swimmers to still be considered clustered. Using this notion of distance, we employ the hierarchical cluster algorithm in Matlab for cluster identification. The number of clusters over time for the two simulations with 1000 individual swimmers are shown in Fig. 1. Individual swimmers are counted as clusters of size one. We see that the number of clusters for the fixed frequency simulation is much lower than that for the simulation where the undulation frequency is allowed to vary stochastically. Additionally, we see that when stochasticity is introduced, cluster growth is halted very early in the simulation.

Effect of film thickness on suspension dynamics

As the flows induced by singular force distributions decay more slowly in 2D than in 3D, we expect the hydrodynamic interactions to play a stronger role as $L_z \to 0$ and, as a result, the instability to exhibit a faster growth rate for thinner films. Figs. 2 and 3 show results from simulations containing 85 swimmers in domains of linear size $L = 4.43d$ and for thicknesses $L_z = 0.277d, 0.554d$, and $1.107d$. The effective area fraction is $\nu = 1.08$ and the swimmers are initially in a polar state. In accordance with the slower formation of

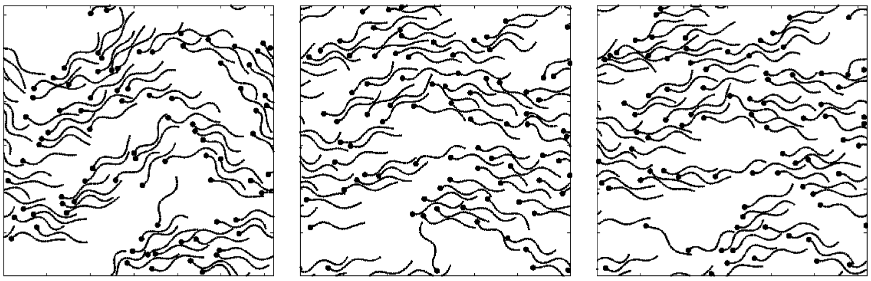

Figure 2: Snapshots at $t = 100T$ of simulations with 85 swimmers all starting from a polar initial state for film thicknesses $L_z = 0.277d, 0.554d, 1.107d$ (left to right).

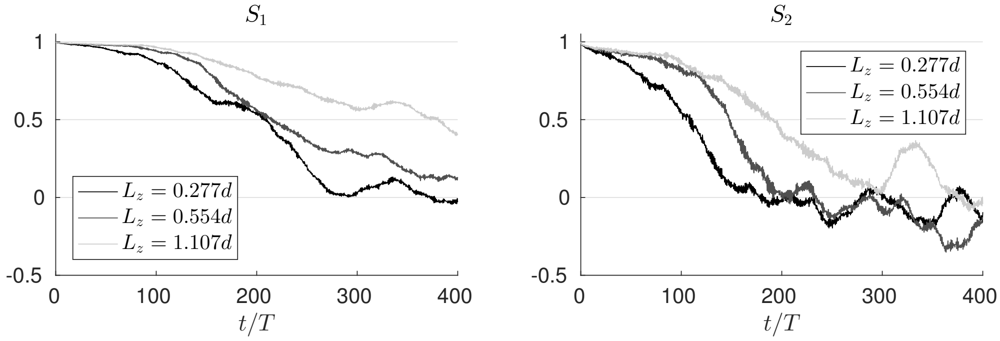

Figure 3: Order parameters as a function of time for the simulations of 85 swimmers in films with thicknesses $L_z = 0.277d, 0.554d$, and $1.107d$. The swimmers are initially aligned with the $-x$ direction.

the bending waves as shown in Fig. 2, the slower decay (see Fig. 3) of the order parameters S_1 and S_2 (as defined in the main text) for thicker films indicate that the shorter range of the hydrodynamic interactions lead to slower growth rates of the instability. The smallest value of L_z used in Fig. 3 matches that for the larger simulations presented in the main text. The order parameter decay rate in those simulations, however, is faster still due to the longer modes afforded by the larger system size.

Effect of undulation frequency variability on aggregation

As mentioned in the main text, low variability in the swimmers' undulation frequencies does not entirely suppress cluster formation and still allows for neighboring swimmers to synchronize and align their waveforms. Snapshots from simulations of a system of size $L = 4.43d$ with 85 initially aligned swimmers and different

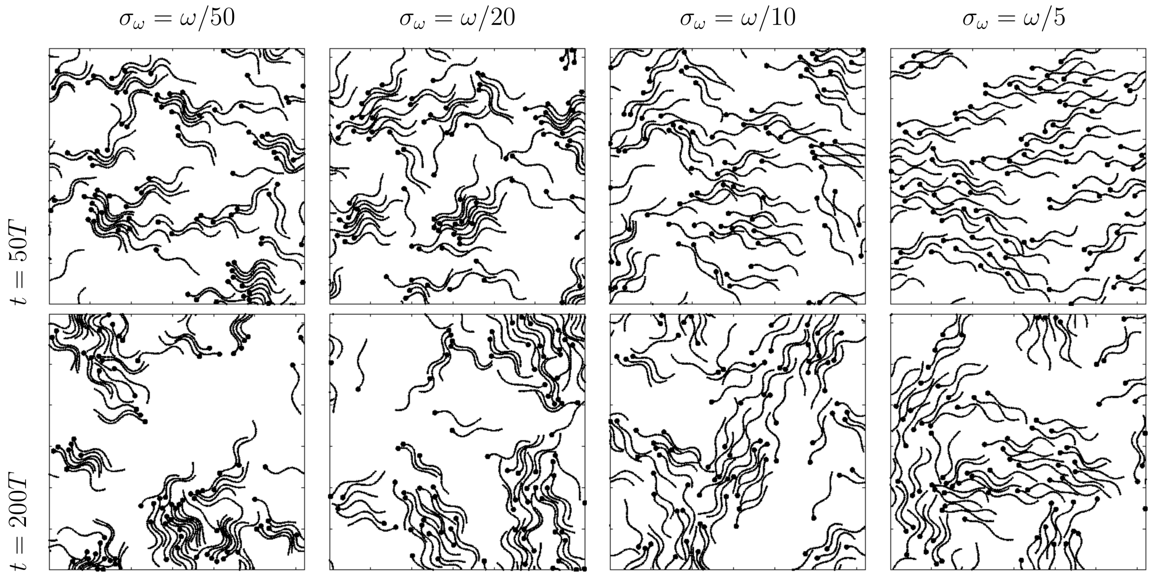

Figure 4: Snapshots at times $t = 50T$ and $t = 200T$ of simulations with 85 initially aligned swimmer with different frequency distributions. Each simulation has domain of size $L = 4.43d$ and film thickness $L_z = 0.277d$. From left to right, the frequency standard deviations are $\sigma_\omega = \omega/50$, $\omega/20$, $\omega/10$, $\omega/5$, while the mean frequency, $\omega = 2\pi/T$, and all other parameters are the same.

values of σ_ω reveal that for small σ_ω, cluster formation persists (see Fig. 4). As σ_ω increases, the number of visually identifiable clusters is reduced and we see instead the emergence of a bending wave.

Tuning the resistive force theory

To remove the hydrodynamic interactions between the swimmers yet still allow for propulsion via undulation, we solve the mobility problem using a drag-based resistive force theory (RFT) [4, 3] rather than FCM. With the drag-based model, the velocity and angular velocity of segment n of a flagellum are related to the force and torque on the segment through

$$\boldsymbol{V}_n = \left(\alpha_{\parallel}\hat{\boldsymbol{t}}_n\hat{\boldsymbol{t}}_n^{\top} + \alpha_{\perp}\left(\boldsymbol{I} - \hat{\boldsymbol{t}}_n\hat{\boldsymbol{t}}_n^{\top}\right)\right)\boldsymbol{F}_n,$$
$$\boldsymbol{\Omega}_n = \beta\boldsymbol{T}_n, \quad \beta = (8\pi b^3\eta)^{-1},$$

where $\alpha_{\parallel}$, $\alpha_{\perp}$, and β are mobility coefficients for the segments. These mobility coefficients are initially estimated based on FCM simulations of straight filaments and subsequently adjusted to ensure that the swimmer waveform and free swimming velocity are comparable to those given by the simulations with FCM, see Fig. 5. For the cell heads, we have for all swimmers m

$$\boldsymbol{V}_m^{(0)} = \gamma\boldsymbol{F}_m^{(0)},$$
$$\boldsymbol{\Omega}_m^{(0)} = \lambda\boldsymbol{T}_m^{(0)},$$

where γ and λ are mobility coefficients taken from FCM simulations of a single spheroid. The numerical values used in the simulations are

$$\gamma = 0.0280(\eta b)^{-1}, \qquad \lambda = 0.002430\eta^{-1}b^{-3},$$
$$\alpha_{\perp} = 0.1100(\eta b)^{-1}, \qquad \alpha_{\parallel} = 0.1700(\eta b)^{-1}.$$

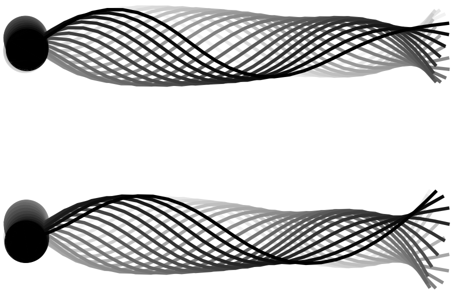

Figure 5: Waveform of an individual swimmer with full hydrodynamics (top) and RFT only (bottom) in the center of mass frame. The swimming direction is to the left and approximately half an undulation period is depicted. The lighter the color of the center-line, the further the snapshot lies in the past.

Energy density spectrum of a randomly forced Stokesian fluid

In this section, we show that a k^{-3} behavior of the fluid energy spectrum can be related to spatially uncorrelated forcing of a 2D fluid. In the following, the Fourier transform and its inverse are defined as

$$\hat{g}(\boldsymbol{k}) = \int_{\mathbb{R}^2} g(\boldsymbol{x}) \mathrm{e}^{-i\boldsymbol{k}\cdot\boldsymbol{x}}\, d^2\boldsymbol{x},$$
$$g(\boldsymbol{x}) = \frac{1}{4\pi^2} \int_{\mathbb{R}^2} \hat{g}(\boldsymbol{k}) \mathrm{e}^{i\boldsymbol{k}\cdot\boldsymbol{x}}\, d^2\boldsymbol{k}\,.$$

We begin by considering a force density, $\boldsymbol{f}(\boldsymbol{x})$, restricted to a square domain,

$$\boldsymbol{f}_L(\boldsymbol{x}) = \boldsymbol{f}(\boldsymbol{x})\, \mathbb{1}_\Omega(\boldsymbol{x}),$$

where $\Omega = [-L/2, L/2] \times [-L/2, L/2]$ and $\mathbb{1}_\Omega$ is the indicator function defined on the set Ω. The Fourier transform of the restricted force density is then

$$\hat{\boldsymbol{f}}_L(\boldsymbol{k}) = \int_{\mathbb{R}^2} \boldsymbol{f}_L(\boldsymbol{x}) \mathrm{e}^{-i\boldsymbol{k}\cdot\boldsymbol{x}}\, d^2\boldsymbol{k}\,.$$

The Fourier transform of the fluid velocity resulting from the restricted force density is given by

$$\hat{\boldsymbol{u}}_L(\boldsymbol{k}) = \frac{1}{\eta k^2} \left(\boldsymbol{I} - \hat{\boldsymbol{k}}\hat{\boldsymbol{k}}^\top\right) \hat{\boldsymbol{f}}_L(\boldsymbol{k})$$

where $(1/\eta k^2)\left(\boldsymbol{I} - \hat{\boldsymbol{k}}\hat{\boldsymbol{k}}^\top\right)$ is the Fourier representation of the inverse Stokes operator, $k = |\boldsymbol{k}|$, and $\hat{\boldsymbol{k}} = \boldsymbol{k}/k$.

The fluid energy density spectrum associated with $\hat{\boldsymbol{u}}_L(\boldsymbol{k})$ is

$$S_L(k) = \frac{k}{2\pi L^2} \int_0^{2\pi} \left\langle |\hat{\boldsymbol{u}}_L(\boldsymbol{k})|^2 \right\rangle d\theta,$$

where θ is related to the two-dimensional wave-vector through $\boldsymbol{k} = (k\cos\theta, k\sin\theta)$ and $\langle\cdot\rangle$ denotes the ensemble average. The energy density spectrum of the infinite system will then be given by $S(k) = \lim_{L\to\infty} S_L(k)$.

As

$$
\begin{aligned}
|\hat{\boldsymbol{u}}_L(\boldsymbol{k})|^2 &= \hat{\boldsymbol{u}}_L(\boldsymbol{k}) \cdot \hat{\boldsymbol{u}}_L(-\boldsymbol{k}) \\
&= \frac{1}{\eta^2 k^4}\left(\boldsymbol{I} - \hat{\boldsymbol{k}}\hat{\boldsymbol{k}}^\top\right) : \left(\hat{\boldsymbol{f}}_L(\boldsymbol{k})\hat{\boldsymbol{f}}_L^\top(-\boldsymbol{k})\right),
\end{aligned}
$$

the fluid energy density spectrum is directly related to the forcing through

$$
S_L(k) = \frac{1}{2\pi\eta^2 k^3 L^2}\int_0^{2\pi}\left(\boldsymbol{I} - \hat{\boldsymbol{k}}\hat{\boldsymbol{k}}^\top\right) : \left\langle \hat{\boldsymbol{f}}_L(\boldsymbol{k})\hat{\boldsymbol{f}}_L^\top(-\boldsymbol{k})\right\rangle d\theta\,.
$$

For $S_L(k) \propto k^{-3}$ to hold, we must have $\left\langle \hat{\boldsymbol{f}}_L(\boldsymbol{k})\hat{\boldsymbol{f}}_L^\top(-\boldsymbol{k})\right\rangle$ independent of k. If, in addition, we require statistically independent and identical force components that do not depend on θ, we have that

$$
\left\langle \hat{\boldsymbol{f}}_L(\boldsymbol{k})\hat{\boldsymbol{f}}_L^\top(-\boldsymbol{k})\right\rangle = AL^2\boldsymbol{I}.
$$

where A is a constant independent of L. The L^2 dependence on the system size guarantees that $S_L(k)$ is well-defined and non-zero as $L \to \infty$ and that the total energy will scale with the system size.

Assuming that the forcing is a statistically stationary spatial process, we may express the correlations of the force density as

$$
\begin{aligned}
&\left\langle \boldsymbol{f}_L(\boldsymbol{x}+\boldsymbol{y})\boldsymbol{f}_L^\top(\boldsymbol{x})\right\rangle \\
&= \frac{1}{L^2}\int_\Omega \left\langle \boldsymbol{f}_L(\boldsymbol{x}+\boldsymbol{y})\boldsymbol{f}_L^\top(\boldsymbol{x})\right\rangle d^2\boldsymbol{x} \\
&= \frac{1}{L^2}\left\langle \int_\Omega \boldsymbol{f}_L(\boldsymbol{x}+\boldsymbol{y})\boldsymbol{f}_L^\top(\boldsymbol{x})\, d^2\boldsymbol{x}\right\rangle.
\end{aligned}
$$

As the force density is strictly restricted to Ω, we may replace the integral over Ω with one over all of $\mathbb{R}^2$. Thus,

$$
\begin{aligned}
&\left\langle \boldsymbol{f}_L(\boldsymbol{x}+\boldsymbol{y})\boldsymbol{f}_L^\top(\boldsymbol{x})\right\rangle \\
&= \frac{1}{L^2}\left\langle \int_{\mathbb{R}^2} \boldsymbol{f}_L(\boldsymbol{x}+\boldsymbol{y})\boldsymbol{f}_L^\top(\boldsymbol{x})\, d^2\boldsymbol{x}\right\rangle,
\end{aligned}
$$

which, from the convolution theorem, may be expressed as

$$
\begin{aligned}
&\left\langle \boldsymbol{f}_L(\boldsymbol{x}+\boldsymbol{y})\boldsymbol{f}_L^\top(\boldsymbol{x})\right\rangle \\
&= \frac{1}{4\pi^2 L^2}\int_{\mathbb{R}^2} \left\langle \hat{\boldsymbol{f}}_L(\boldsymbol{k})\hat{\boldsymbol{f}}_L^\top(-\boldsymbol{k})\right\rangle \mathrm{e}^{i\boldsymbol{k}\cdot\boldsymbol{y}}\, d^2\boldsymbol{k}\,.
\end{aligned}
$$

Finally, using the expression for $\left\langle \hat{\boldsymbol{f}}_L(\boldsymbol{k})\hat{\boldsymbol{f}}_L^\top(-\boldsymbol{k})\right\rangle$ given above, we see that

$$
\begin{aligned}
\left\langle \boldsymbol{f}_L(\boldsymbol{x}+\boldsymbol{y})\boldsymbol{f}_L^\top(\boldsymbol{x})\right\rangle &= \frac{AL^2\boldsymbol{I}}{4\pi^2 L^2}\int_{\mathbb{R}^2} \mathrm{e}^{i\boldsymbol{k}\cdot\boldsymbol{y}}\, d^2\boldsymbol{k} \\
&= A\boldsymbol{I}\delta(\boldsymbol{y})
\end{aligned}
$$

for any L. Allowing $L \to \infty$ then yields

$$
\left\langle \hat{\boldsymbol{f}}(\boldsymbol{x}+\boldsymbol{y})\hat{\boldsymbol{f}}^\top(\boldsymbol{x})\right\rangle = A\boldsymbol{I}\delta(\boldsymbol{y})
$$

and we see that a k^{-3} decay in the fluid energy density spectrum is given by uncorrelated spatial forcing of the fluid.

References

[1] U. M. Ascher and L. R. Petzold. *Computer methods for ordinary differential equations and differential-algebraic equations*. Vol. 61. SIAM, 1998. doi: 10.1137/1.9781611971392.

[2] C. G. Broyden. "A Class of Methods for Solving Nonlinear Simultaneous Equations". In: *Math. Comp.* 19.92 (1965), pp. 577–593. doi: https://doi.org/10.1090/S0025-5718-1965-0198670-6.

[3] R. E. Johnson and C. J. Brokaw. "Flagellar Hydrodynamics – A comparison between Resisitive-Force Theory and Slender-Body Theory". In: *Biophys. J.* 25 (1979), pp. 113–127. doi: http://dx.doi.org/10.1016/S0006-3495(79)85281-9.

[4] E. Lauga and T. R. Powers. "The hydrodynamics of swimming microorganisms". In: *Rep. Prog. Phys.* 72.9 (2009), p. 096601. doi: 10.1088/0034-4885/72/9/096601.

[5] D. Liu, E. Keaveny, M. Maxey, and G. Karniadakis. "Force-coupling method for flows with ellipsoidal particles". In: *J. Comput. Phys.* 228.10 (2009), 3559–3581. doi: 10.1016/j.jcp.2009.01.020.

[6] S. Lomholt and M. R. Maxey. "Force-coupling method for particulate two-phase flow: Stokes flow". In: *Journal of Computational Physics* 184.2 (2003), pp. 381 –405. doi: https://doi.org/10.1016/S0021-9991(02)00021-9.

[7] M. Maxey and B. Patel. "Localized force representations for particles sedimenting in Stokes flow". In: *International Journal of Multiphase Flow* 27.9 (2001), pp. 1603 –1626. doi: https://doi.org/10.1016/S0301-9322(01)00014-3.

[8] Y. Yang, J. Elgeti, and G. Gompper. "Cooperation of sperm in two dimensions: Synchronization, attraction, and aggregation through hydrodynamic interactions". In: *Phys. Rev. E* 78.061903 (2008). doi: 10.1103/physreve.78.061903.
