# Supplementary figures and images for: From flagellar undulations to collective motion: predicting the dynamics of sperm suspensions
Source: J R Soc Interface. 2018 Mar 21;15(140):20170834. doi: 10.1098/rsif.2017.0834 (PMC5908526; doi:10.1098/rsif.2017.0834)

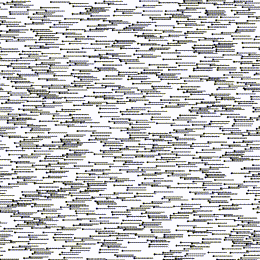

Supplement: Flagellar undulations in a suspension synchronized sperm [file rsif20170834supp2.gif]

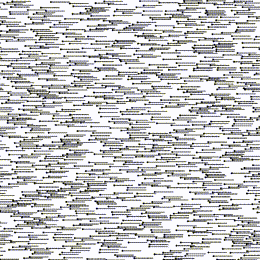

Supplement: Aggregation in a synchronized sperm suspension [file rsif20170834supp3.gif]

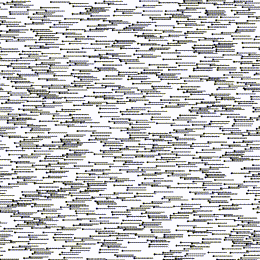

Supplement: Large-scale motion in a sperm suspension [file rsif20170834supp4.gif]

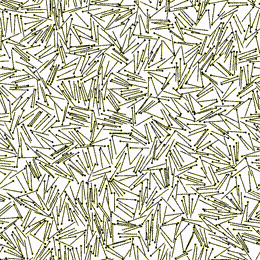

Supplement: Evolution of an initially isotropic sperm suspension in the absence of hydrodynamics interactions [file rsif20170834supp5.gif]

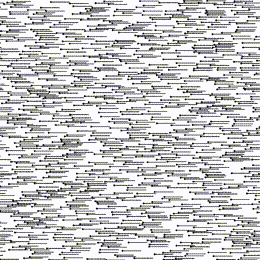

Supplement: Evolution of an initially polar sperm suspension in the absence of hydrodynamics interactions [file rsif20170834supp6.gif]

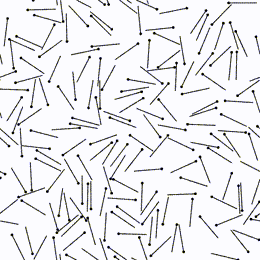

Supplement: Dynamics of an initially isotropic low-density sperm suspension [file rsif20170834supp7.gif]

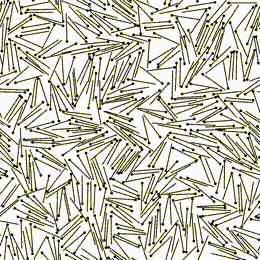

Supplement: Dynamics of an initially isotropic high-density sperm suspension [file rsif20170834supp8.gif]
